# The Implications in Meat Quality and Nutrition by Comparing the Metabolites of Pectoral Muscle between Adult Indigenous Chickens and Commercial Laying Hens

**DOI:** 10.3390/metabo13070840

**Published:** 2023-07-11

**Authors:** Lingqian Yin, Li Liu, Yuan Tang, Qian Chen, Donghao Zhang, Zhongzhen Lin, Yan Wang, Yiping Liu

**Affiliations:** Farm Animal Genetic Resources Exploration and Innovation Key Laboratory of Sichuan Province, Sichuan Agricultural University, Chengdu 611130, China

**Keywords:** non-targeted metabonomics, UHPLC-MS/MS, pectoral muscle, chicken, lipid metabolism

## Abstract

Aged chickens are often a secondary dietary choice, owing to the poor organoleptic qualities of their meat. With regard to the meat quality of chickens, the metabolic profiles of pectoral muscle in Guangyuan grey chickens (group G) and Hy-Line grey hens (group H) aged 55 weeks were compared via ultra-high-performance liquid chromatography–tandem mass spectrometry (UHPLC-MS/MS). A total of 74 metabolites were identified with differential changes in the ion model. Lipids and lipid-like molecules comprised the largest proportion among the different metabolites. The content of myristic acid and palmitic acid were found to be higher in the pectoral muscle of group G, while group H showed significantly higher levels of glycerophospholipid molecules, such as LPC(18:2/0:0), Pi(38:5), Pc(16:0/16:0), and Pe(16:1e/14-hdohe). KEGG pathway analysis indicated that the abundant metabolites in group G were mainly involved in energy metabolism and fatty acid biosynthesis and metabolism, whereas those of group H were mainly attributed to the metabolism of unsaturated fatty acids and amino acids. Overall, the differences in lipid and amino acid metabolism in pectoral muscle appear to be responsible for the difference in meat quality between indigenous chickens and commercial laying hens.

## 1. Introduction

Poultry meat and eggs, particularly chicken, a high-quality and readily available source of animal protein, are consumed in vast qualities on a global scale. According to the latest FAO statistics [1], the average intake of eggs and poultry meat in 2020 was 10.45 and 16.4 kg/capita/year, respectively. Among the common livestock animals (cattle, pigs, and chickens), the consumption of poultry meat is the highest. To meet the multiple requirements for poultry products, the commercial line of chickens for meat and eggs has been bred since the 1920s. Most broilers are raised for short periods. The market age of broilers is about 6 weeks. Laying hens are raised until the end of the laying cycle. Although they perform better in protein content, spent laying hens enter the market at a low price for animal feed and human food processing [2,3], or are even disposed of as fertilizer and waste, which may reduce farming efficiency and harm the environment.

Spent laying hens are generally not accepted by consumers, mainly due to poor organoleptic properties. A typical characteristic of old chickens is a high content of collagen, which leads to an increase in shear force, and subsequently, tougher meat [4]. However, there is other economic value in spent chicken. As an animal protein product, the protein content of spent chicken meat is not inferior to that of broilers, and it even has a superior composition of essential amino acids [5]. A variety of bioactive substances were also confirmed to be enriched in spent laying hens [6,7]. Many value-added processes have been developed to improve the utilization of spent chicken over the past decades, such as in functional foods [8,9] and biomaterials [10,11]. Among these processes, taking full advantage of spent chickens as a source of food can be both convenient and environmentally friendly, particularly during times of food shortage.

Metabolomics is a science that studies the types, quantities, and dynamic changes of metabolites (endogenous metabolites) caused by external stimuli, pathophysiological changes, and genetic mutations of organisms [12]. It can reflect the physiological state of organisms more directly and accurately compared with transcriptome and proteomics. Since the 21st century, metabolomics has been widely used in pharmaceuticals [13], clinical applications [14], food [15] and environmental sciences [16], agriculture [17], and animal husbandry [18]. Non-targeted metabonomic technology is a method which detects various metabolites in unbiased, large-scale samples using ultra-high-performance liquid chromatography–tandem mass spectrometry (UHPLC-MS/MS).

Due to differences in dietary culture, attitudes towards aged chicken vary among people between regions. Consumers in some countries prefer the meat of older chickens; however, it also depends on the breed. In China, consumers prefer aged indigenous chickens instead of aged commercial breeds. This consumption habit leads to the phenomenon that the value of indigenous chicken is much higher than the commercial line, even though they are of the same age. The Guangyuan grey chicken is an indigenous meat- and egg-type breed, which goes on sale at the age of 180 d with a weight of around 1.78 kg. However, a portion of these hens are raised until they are 52 weeks of age, as their primary products are considered to be nutraceuticals, due to the associations of its grey plumage in local culture. The Hy-Line grey layer is a commercial hybridized layer strain, which performs very well in egg production. The end of the laying cycle of Hy-Line layers occurs at approximately 80 weeks; then, they are classed as aged chickens. Thus, the aim of the present study is to compare the various metabolites in the pectoral muscles of adult Guangyuan grey chickens and Hy-Line grey hens using UHPLC-MS/MS. The results could have implications for metabolism regulation, meat quality, and nutritional profile between indigenous chickens and commercial laying hens.

## 2. Materials and Methods

### 2.1. Birds and Sample Preparation

The animals for this trial were raised in Sichuan Tianguan Ecological Agriculture and Husbandry Co., Ltd. (Guangyuan, Sichuan, China). Two hundred one-day-old female Guangyuan grey chickens (Group G) and Hy-Line grey chickens (Group H) were reared on the floor in the closed room. All birds had free access to commercial brood feed (Guangyuan Zhuangniu Agriculture and Animal Husbandry Technology Co., Ltd., Guangyuan, Sichuan, China) and water. At six weeks of age, 90 Guangyuan grey chickens and 90 Hy-Line grey chickens were selected randomly for this trial. The birds of each breed were distributed into 6 replicate groups, with each group comprising 15 birds. Each bird was transferred to a single cage with ad libitum feed (Table 1) and water. During the trial, the temperature was set to 35 °C for first three days, then 28–32 °C from four days to three weeks, and then set to 26 °C from four weeks on. The humidity was set to 70% for first two weeks, 55% for three to six weeks, and 45% from then on. The light program was set to 24L:0D for the first week, 8L:16D from 2 to 20 weeks, 14L:10D from 21 weeks until the end of laying cycle, with one hour of light added every week until it reached 16L:8D. The intensity of light was 5 lux for first two weeks. An LED light source (5 watts) was installed every 3 m, 2.5 m above the ground for rearing stage. At the age of 55 weeks, six birds with similar weight (around 1.74 ± 0.22 kg) from each breed were selected to collect pectoral major muscles. Samples for histology were cut parallel to the muscle fibers in a rectangular form (1 cm in length and width) at an optimal cutting temperature for compound embedding and then frozen in dry ice. After removing the connective tissue, the samples for metabolite detection were snap-frozen in liquid nitrogen.

### 2.2. UHPLC-MS/MS

#### 2.2.1. Sample Preparation

Six snap-frozen pectoral muscle samples from each breed were used to determine metabolite profile. Briefly, about 5 mg of each sample was taken and pulverized. Then, we added it into the mix solution of methanol/acetonitrile/water (Sinopharm, Beijing, China). After vortices, centrifugation, and drying, the mix solution of acetonitrile/water was added to the tube. After centrifugation, supernatant was used for UHPLC-MS/MS detection.

#### 2.2.2. UHPLC-MS/MS Analysis

The samples were first analyzed using a UHPLC (1290 Infinity LC, Agilent Technologies, Santa Clara, CA, USA) coupled to a quadrupole time-of-flight (AB Sciex TripleTOF 6600, Boston, MA, USA). The supernatant was added in a precooled 2.1 mm × 100 mm ACQUITY UPLC BEH Amide 1.7 μm column (Waters, Wexford, lreland) for HILIC separation. The mobile phase contained A (25 mM ammonium acetate and 25 mM ammonium hydroxide in water) (XilONG SCIENTIFIC, Guangdong, China) and B (acetonitrile) (Sinopharm, Beijing, China). After linearly reduced gradient elution from 95% B to 40% B about 9 min, then increased to 95% in 6 s, with a 3 min re-equilibration period employed. After separation, the samples were analyzed with Triple TOF 6600 mass spectrometer (AB SCIEX, Boston, MA, USA), and the primary and secondary spectra were collected through electrospray ionization (ESI) positive and negative ionization modes.

### 2.3. Oil-Red O Staining and Triglyceride Measurement

The frozen samples were serial sectioned into 6 μm slices and fixed in 10% neutral buffered formalin fixative (Solarbio, Beijing, China) for 10 min. The slices were washed with precooled distilled water (Sangon Biotech, Beijing, China) for 2 min and immersed in isopropyl alcohol (Sinopharm, China) for 2 s. Then, the slices were stained for 15 min with oil red O solution (Sangon Biotech, China). The slices were immersed in isopropyl alcohol again for 2 s and rinsed under precooled distilled water for 2 min. Mayer hematoxylin (Sangon Biotech, Beijing, China) was used to stain the cell nucleus for 30 s and sealed with glycerol jelly mounting medium (XilONG SCIENTIFIC, Guangdong, China). The slices were observed and photographed under an inverted microscope (BX53F, Olympus, Tokyo, Japan). The triglyceride (TG) content of pectoral muscle samples was measured using the glycerol-3-phosphate oxidase and peroxidase method using commercial kits purchased from Nanjing Jiancheng Bioengineering Institute (Jiangsu, China).

### 2.4. Statistical Analysis

The data were submitted to a normality and homogeneity of variance test first. The R package ropls [19,20] was used for subsequent multivariate analysis, including Pareto-scaled principal component analysis (PCA) and orthogonal partial least-squares discriminant analysis (OPLS-DA). Seven-fold cross-validation and response permutation testing were used to evaluate the robustness of the model. Student’s *t* test was applied to determine the statistical significance between two groups of independent samples at a 5% confidence level (*p* < 0.05). OPLS-DA VIP > 1 and *p* < 0.05 were used to screen significant changed metabolites. Pearson’s correlation analysis was performed to determine the correlation between two variables.

## 3. Results and Discussion

### 3.1. Metabolomic Composition of Guangyuan Grey Chicken and Hy-Line Grey Hen

Based on bioinformatics analysis of non-targeted metabonomic data, 889 metabolites were identified in the pectoral muscles of Guangyuan grey hens and Hy-Line grey hens. The chemical classification of these metabolites is shown in Table 2. Lipids and lipid-like molecules comprised the largest proportion of the metabolites. It indicated that lipid metabolism was active in the pectoral muscle of aged chickens. The lipid molecules of two breeds were further analyzed. The most numerous of these lipid molecules were glycerophospholipids. The same result was observed in a study by Li et al. [21], which found that glycerophospholipids were the dominant class among the lipid molecules identified in the breast muscle. Glycerophospholipids have high structural diversity and participate in many physiological processes in animals, such as the synthesis of biofilm [22], oxidative phosphorylation [23], and signal transduction [24]. They may be involved in muscle cell fusion, smooth muscle contraction, and oxidative phosphorylation in pectoral muscle.

### 3.2. Screening and Correlation Analysis of Differential Metabolites

**Figure 1:**
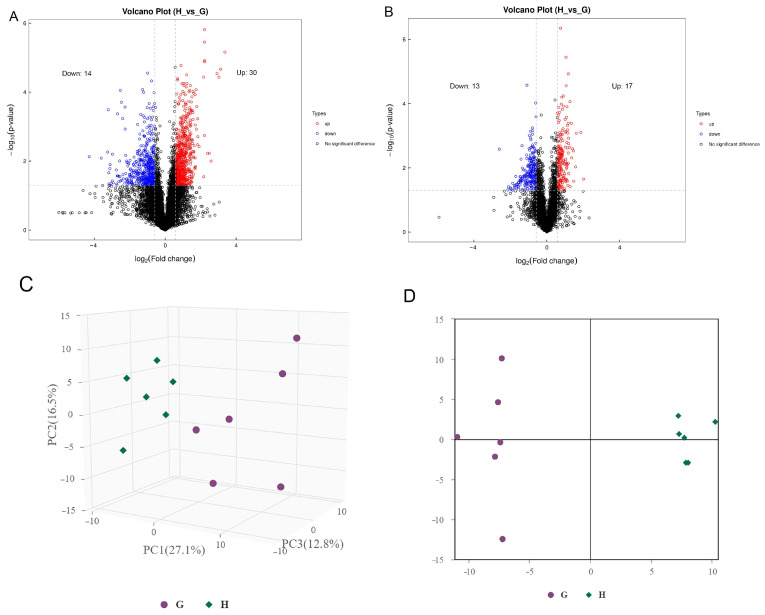
The different metabolite analysis between groups. (**A**) Volcanic plot of metabolites in the positive ion modes. (**B**) Volcanic plot of metabolites in the negative ion modes. Red circle, upregulated significantly differential metabolites; blue circle, down-regulated significantly differential metabolites. (**C**) PCA scores of group H and G. (**D**) OPLS-DA scores of group H and G.

**Figure 2:**
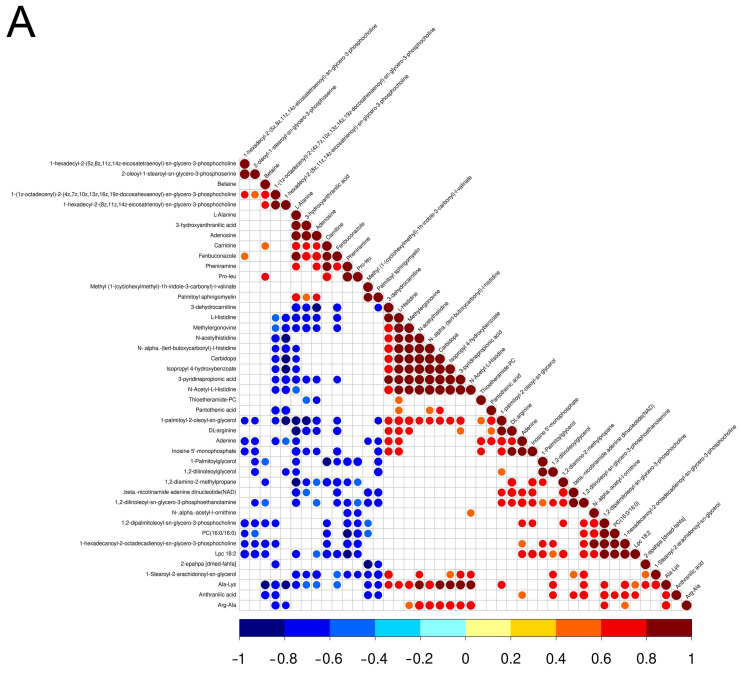

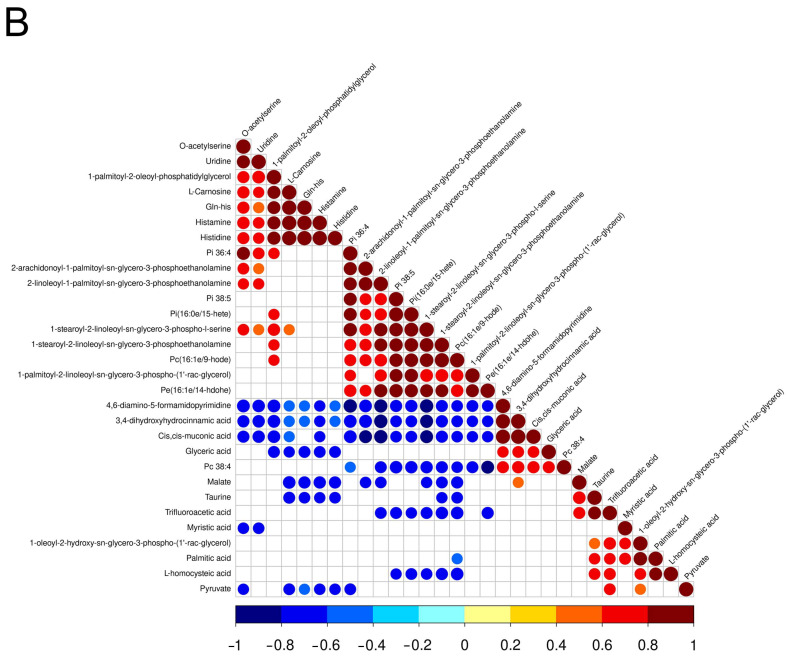
Heat maps of correlation between differential metabolites in the positive (**A**) and negative (**B**) ion modes. Red, positive correlation; blue, negative correlation.

The market value of aged chickens is low due to their poor sensory traits, especially aged laying hens [25]. However, people from some areas prefer aged hens for stews because they believe the aged chickens have higher nutritional value and flavor, which results in a high market value of aged hens. Previous studies confirmed that there are differences in meat quality between aged laying hens and broilers, for instance, water holding capacity, tenderness, and cooking loss [26]. In addition, there were differences in nutritional value and flavor between laying hens and broilers [5,27]. To further reveal the metabolic reasons for the differences in meat quality between aged laying hens and broilers, UHPLC-MS/MS was employed to determine the metabolites of the pectoral muscle of aged laying hens and broilers. A total of 74 differential metabolites were identified in the positive and negative ion model (Table 3), including 27 metabolites higher in group G and 47 metabolites higher in group H (Figure 1A,B). The results of PCA and OPLS-DA also indicated that a significant difference was found in the metabolism of pectoral muscle between group H and group G (Figure 1C,D). The result of Pearson correlation analysis showed that 44 metabolites were correlated in the positive ion modes and 30 metabolites were correlated in the negative ion modes (Figure 2A,B).

Lipids and lipid-like molecules comprised the largest proportion of the different metabolites. Among these lipids and lipid-like molecules, the content of myristic acid and palmitic acid in the pectoral muscle of group G was higher than that of group H. In the correlation analysis, there was a correlation between myristic and palmitic acid (R = 0.43). As saturated fatty acids, myristic acid and palmitic acid were reported to be linked with adverse cardiovascular disease [28,29]. However, myristic acid was also widely used as a flavor component in the food industry with a waxy taste [30], which may contribute to the unique flavor and taste of group G. Meanwhile, some substances involved in energy metabolism were also found to be higher in group G, such as carnitine, pyruvate, and malate. Carnitine, the key component of the carnitine shuttle system, was discovered to participate in long-chain fatty acid transportation to the mitochondria for further oxidation [31]. Pyruvate, as the final product of glycolysis, participates in the process of aerobic and anaerobic oxidation and the oxidative and synthesis of lipids [32,33]. These clues indicated that the energy metabolism may be more active in the pectoral muscle of group G. Malate, the intermediate of the tricarboxylic acid (TCA) cycle, which was also higher in the pectoral muscle of group G. Bio-fermented malic acid supplemented in meal affected the water-holding capacity of breast muscle of broilers [34]. In pigs, a diet supplemented with L-malic acid could improve the meat color and carcass traits of finishing pigs [35]. The higher content of malate found in group G may affect the meat quality of the pectoral muscle.

A dozen intermediates of lipid synthesis and metabolism were found to be higher in group H, such as 1-palmitoyl-2-oleoyl-sn-glycerol, 1,2-dipalmitoleoyl-sn-glycero-3-phosphocholine, and 1-Stearoyl-2-arachidonoyl-sn-glycerol, suggesting that the synthesis and metabolism of lipids were active in the muscle of aged laying hens. In the egg yolk, glycerophospholipids were found to be the most abundant lipids [36]. In this study, some glycerophospholipid molecules were also observed to be significantly higher in the pectoral muscle of group H, such as LPC(18:2/0:0), Pi(38:5), Pi(16:0e/15-hete), Pi(36:4), Pc(16:1e/9-hode), Pc(16:0/16:0), and Pe(16:1e/14-hdohe). LPC and phosphatidylinositol have been confirmed to be closely related to biofilm formation and lipid transport [37,38,39]. These results indicated that the glycerophospholipids existing in the pectoral muscle of laying hens may be not only involved in the metabolism of muscle cells, but also serve as a store for egg formation.

In this study, some amino acids, dipeptides and amino acid derivatives were found to be higher in group G and H. L-Alanine, L-homocysteic acid, and Pro-leu were higher in group G. Previous research has found that slow-growing chickens developed a specific appetite for Ala, which may be due to the alanine-derived pyruvate used as an energy source to compensate for the lack of glucose-derived pyruvate [40]. As a slow-growing indigenous chicken, L-Alanine and pyruvate were found to be higher in group G, which indicated alanine may be involved in energy supply in the pectoral muscle of Guangyuan grey chickens. The contents of histidine, DL-arginine, Ala-Lys, Gln-his, L-Carnosine, and *N*-acetylhistidine were higher in group H. Chicken meat contains a large amount of histidine-containing dipeptides, which were confirmed to be beneficial for health [41]. Carnosine is a dipeptide composed of α-alanine and L-histidine. Notably, previous studies have revealed the therapeutic potential of carnosine in several diseases, such as neurological disorders [42], osteoporosis [43], peripheral vascular disease [44], and aging [45]. Another study revealed that a higher content of carnosine in diet can improve chicken meat quality, including pH_45 min_, shear force, and drip loss [46]. Hence, carnosine supplementation in the diet during the later life stage of aged laying hens may improve their meat quality and increase the carnosine content in the meat, which may make the aged laying hens serve as a potential food source of carnosine.

### 3.3. KEGG Pathway Analysis of Significantly Differential Metabolites

Through KEGG analysis, 74 differential metabolites were enriched to 64 KEGG pathways. There were 23 and 27 KEGG pathways unique to group H and group G, respectively. Among the pathways of group H, three pathways were related with unsaturated fatty acid metabolism, and five pathways of group H were involved in amino acid metabolism (Table 4). Interestingly, the GnRH signaling pathway was also found in the enrichment pathway of group H, which only contained 1-Stearoyl-2-arachidonoyl-sn-glycerol, also known as diacylglycerol(18:0/20:4). Diacylglycerol is a second messenger in the GnRH signaling pathway, which plays an important role in activating downstream calcium ions and the PKC signaling pathway [47]. This metabolite was also enriched in the adipocytokine signaling pathway of group H. Previous studies found that the GnRH signaling pathway was involved in fat deposition and lipid metabolism in the breast muscle of chicken and duck [48,49]. The metabolites with a correlation coefficient greater than 0.7 (|R| > 0.7) with diacylglycerol were 1,2-dilinoleoyl-sn-glycero-3-phosphoethanolamine, 3-dehydrocarnitine, Ala-Lys, adenosine, L-Alanine and palmitoyl sphingomyelin. Among the above metabolites, 1,2-dilinoleoyl-sn-glycero-3-phosphoethanolamine, 3-dehydrocarnitine, and palmitoyl sphingomyelin may be involved in the process of fat deposition and lipid metabolism regulated by diacylglycerol(18:0/20:4). Overall, it was conjectured that diacylglycerol may be a key factor in mediating the GnRH signaling pathway and participating in the processes of fat deposition and lipid metabolism in the pectoral muscles of chicken after sexual maturity.

In group G, six pathways associated with energy metabolism and six pathways associated with fatty acid biosynthesis and metabolism were identified (Table 4). These results indicate that the energy and fatty acid biosynthesis and metabolism between aged layers and broilers may be different. The glycolysis of muscle after slaughter has been deemed to have a great influence on meat quality, such as pH, color, and drip loss [50,51]. Glycolysis directly induces the breakdown of muscle glycogen and the accumulation of lactic acid, thereby altering muscle pH [52]. The significantly enriched glycolysis/gluconeogenesis pathway in Group G contains one differential metabolite, pyruvate, which was found to be higher in the pectoral muscle of group G. It was presumed that the pH of group G may be different compared with group H. In group G, six pathways associated with fatty acid biosynthesis and metabolism were determined in pectoral muscle. The biosynthesis and metabolism of fatty acid were coordinated in cells [53]. Fatty acid biosynthesis and metabolism were both found to be active in group G, which may indicate that the synthesis and utilization of lipids were more active in aged Guangyuan grey chickens. This was consistent with the characteristic of broilers that less lipids are stored for egg production.

### 3.4. Oil Red O Staining and Triglyceride Measurement of Pectoral Muscle

**Figure 3:**
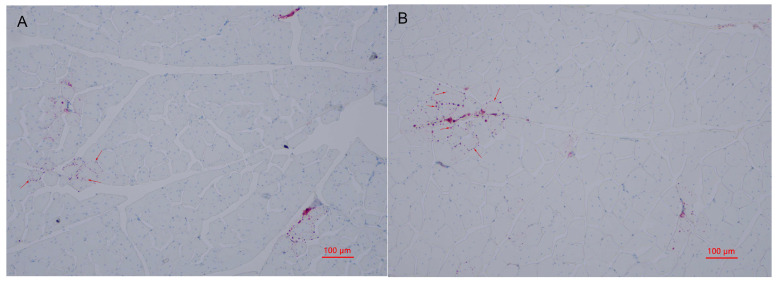
Oil red O staining of pectoral muscle sections of Guangyuan grey chicken (**A**) and Hy-Line grey hen (**B**). The sample was observed with an optical microscope (100×). Red arrows point to lipid droplets.

**Figure 4:**
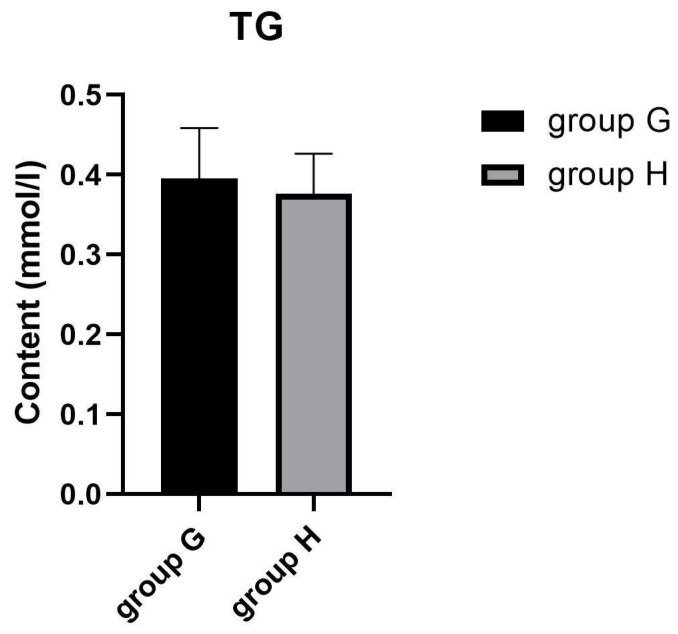
The content of triglyceride in group G and H.

Many metabolites involved in lipid metabolism were identified in groups G and H which may be involved in the regulation of intramuscular fat deposition. The contents of intramuscular fats are important indexes to evaluate meat quality, since they were confirmed to affect the flavor and tenderness qualities of chicken meat [54]. In this study, oil red O staining was employed to observe the distribution of lipid droplets in muscle cells. As shown in Figure 3A,B, the lipid droplets are mainly distributed in cell membranes and intercellular space in groups G and H. Compared with group G, more intracellular lipid droplets were in the pectoral muscles of group H. These results indicate that the lipid deposition of pectoral muscles between laying hens and broilers may be different, which may be to adapt to the different production modes of laying hens and broilers. TG in skeletal muscle was confirmed to be decisive for IMF deposition [54]. Herein, we determined the content of triglyceride (TG) in the pectoral muscles of group G and H. The result showed that there was no significant difference of TG content between group G and H (Figure 4). These results suggested that older laying hens may exhibit similar flavor and tenderness compared with female broilers under the same feeding conditions.

## 4. Conclusions

The metabolite profiles of the pectoral muscle of aged Guangyuan grey chicken and Hy-Line grey laying hens were determined using UHPLC-MS/MS. A total of 74 differential metabolites were identified in the positive and negative ion model. Lipids and lipid-like molecules accounted for the largest proportion among the different metabolites. The content of myristic acid and palmitic acid was higher in the pectoral muscle of group G. Some substances involved in energy metabolism were found to be higher in group G, such as carnitine, pyruvate, and malate. These metabolites which were higher in group G were mainly enriched in glycolysis/gluconeogenesis, citrate cycle (TCA cycle), and fatty acid biosynthesis and degradation. Glycerophospholipid molecules were observed to be higher in the pectoral muscle of group H, such as LPC(18:2/0:0), Pi(36:4), Pc(16:0/16:0), and Pe(16:1e/14-hdohe). The contents of histidine, DL-arginine, Ala-Lys, Gln-his, L-Carnosine, and *N*-acetylhistidine were found to be higher in group H. These metabolites which were higher in group H were mainly enriched in glycerophospholipid metabolism, linoleic acid metabolism, the calcium signaling pathway, and beta-alanine metabolism. In addition, the GnRH signaling pathway was also found in group H, which proved the close connection between reproduction and metabolic processes.

## Figures and Tables

**Table 1:** Composition of diet and nutrition content.

Ingredient (%)	Rearing Stage (%)	Laying Stage (%)
Corn	63.00	59.80
Soybean meal (sol.)	11.50	12.80
Maize gluten meal	3.00	5.00
Wheat bran	7.00	4.40
Rapeseed meal (sol.)	8.00	7.00
Soybean oil	2.80	3.30
Dicalcium phosphate	1.20	1.00
Limestone	1.00	4.20
Premixes ^a^	2.50	2.50
Nutrition content		
ME (MJ/Kg)	12.15	12.14
CP (%)	16.22	17.01
Calcium (%)	0.85	2.00
Available P (%)	0.4	0.34
Lys (%)	0.64	0.66
Met (%)	0.29	0.31

^a^ Premix included the following per kg of feed: Vitamin A ≥ 390 KIU, Vitamin D3 ≥ 150 KIU, Vitamin E ≥ 1240, Vitamin B ≥ 185, Vitamin B2 ≥ 260, Vitamin B6 ≥ 160, Vitamin B12 ≥ 1.2, Vitamin K3 ≥ 100, D-biotin ≥ 12, D-pantothenic acid ≥ 470, folic acid ≥ 57, niacin ≥ 1660, hydrogenated choline ≥ 15,000, methionine ≥ 43,000, Fe ≥ 2000, Cu ≥ 380, Mn ≥ 3900, Zn ≥ 2800, I ≥ 25, Se ≥ 10.

**Table 2:** The chemical classification of metabolites.

Classfication	Percent (%)
Lipids and lipid-like molecules	31.16
Organic acids and derivatives	25.98
Undefined	10.57
Organoheterocyclic compounds	8.10
Organic oxygen compounds	7.76
Benzenoids	5.62
Nucleosides, nucleotides, and analogues	4.50
Organic nitrogen compounds	3.04
Phenylpropanoids and polyketides	2.36
Alkaloids and derivatives	0.68
Homogeneous non-metal compounds	0.112
Organosulfur compounds	0.112

**Table 3:** Differential metabolites identified in the pectoral muscles of group G and group H.

Name	SuperClass	Fold Change	FC Type	*p*-Value	VIP
Negative ion mode					
Pi 38:5	Lipids and lipid-like molecules	3.07	up	<0.05	2.57
1-palmitoyl-2-oleoyl-phosphatidylglycerol	Lipids and lipid-like molecules	2.99	up	<0.05	4.66
Gln-his	Organic acids and derivatives	2.15	up	<0.05	1.01
Pi(16:0e/15-hete)	Lipids and lipid-like molecules	2.15	up	<0.05	1.17
2-linoleoyl-1-palmitoyl-sn-glycero-3-phosphoethanolamine	Lipids and lipid-like molecules	2.15	up	<0.05	1.73
Pc(16:1e/9-hode)	Lipids and lipid-like molecules	2.09	up	<0.05	5.19
1-stearoyl-2-linoleoyl-sn-glycero-3-phospho-l-serine	Lipids and lipid-like molecules	2.08	up	<0.05	1.57
1-stearoyl-2-linoleoyl-sn-glycero-3-phosphoethanolamine	Lipids and lipid-like molecules	1.87	up	<0.05	1.56
2-arachidonoyl-1-palmitoyl-sn-glycero-3-phosphoethanolamine	Lipids and lipid-like molecules	1.86	up	<0.05	3.09
Histidine	Organic acids and derivatives	1.74	up	<0.05	4.42
Histamine	Organic nitrogen compounds	1.72	up	<0.05	1.79
Pe(16:1e/14-hdohe)	Lipids and lipid-like molecules	1.68	up	<0.05	1.16
L-Carnosine	Organic acids and derivatives	1.65	up	<0.05	23.99
O-acetylserine	Organic acids and derivatives	1.65	up	<0.05	1.02
Pi 36:4	Lipids and lipid-like molecules	1.55	up	<0.05	1.16
Uridine	Nucleosides, nucleotides, and analogues	1.48	up	<0.05	2.52
1-palmitoyl-2-linoleoyl-sn-glycero-3-phospho-(1′-rac-glycerol)	Lipids and lipid-like molecules	1.47	up	<0.05	1.11
L-homocysteic acid	Organic acids and derivatives	0.86	down	<0.05	3.44
Palmitic acid	Lipids and lipid-like molecules	0.82	down	<0.05	6.56
4,6-diamino-5-formamidopyrimidine	Organoheterocyclic compounds	0.80	down	<0.05	1.24
Myristic acid	Lipids and lipid-like molecules	0.77	down	<0.05	1.06
Taurine	Organic acids and derivatives	0.73	down	<0.05	5.10
Pc 38:4	Undefined	0.68	down	<0.05	1.11
1-oleoyl-2-hydroxy-sn-glycero-3-phospho-(1′-rac-glycerol)	Lipids and lipid-like molecules	0.67	down	<0.05	1.07
Malate	Organic acids and derivatives	0.61	down	<0.05	2.14
Trifluoroacetic acid	Organic acids and derivatives	0.55	down	<0.05	1.32
Pyruvate	Organic acids and derivatives	0.55	down	<0.05	7.65
Glyceric acid	Organic oxygen compounds	0.55	down	<0.05	1.02
3,4-dihydroxyhydrocinnamic acid	Undefined	0.54	down	<0.05	1.10
Cis,cis-muconic acid	Lipids and lipid-like molecules	0.51	down	<0.05	5.76
Positive ion mode					
Ala-Lys	Organic acids and derivatives	4.65	up	<0.05	1.96
Carbidopa	Phenylpropanoids and polyketides	2.65	up	<0.05	3.14
β-nicotinamide adenine dinucleotide(NAD)	Undefined	2.44	up	<0.05	1.08
Adenine	Organoheterocyclic compounds	2.30	up	<0.05	1.21
1-palmitoyl-2-oleoyl-sn-glycerol	Lipids and lipid-like molecules	2.28	up	<0.05	1.15
2-epahpa [dmed-fahfa]	Undefined	2.19	up	<0.05	1.58
1,2-dipalmitoleoyl-sn-glycero-3-phosphocholine	Lipids and lipid-like molecules	2.18	up	<0.05	2.46
3-pyridinepropionic acid	Undefined	2.16	up	<0.05	1.28
Arg-Ala	Organic acids and derivatives	2.11	up	<0.05	2.26
1,2-dilinoleoylglycerol	Lipids and lipid-like molecules	2.09	up	<0.05	1.12
Isopropyl 4-hydroxybenzoate	Benzenoids	2.07	up	<0.05	1.71
Anthranilic acid	Benzenoids	2.06	up	<0.05	1.54
Lpc 18:2	Lipids and lipid-like molecules	2.00	up	<0.05	3.70
DL-arginine	Organic acids and derivatives	1.99	up	<0.05	1.29
1,2-diamino-2-methylpropane	Organic nitrogen compounds	1.97	up	<0.05	1.09
Methylergonovine	Alkaloids and derivatives	1.95	up	<0.05	1.06
*N*-acetylhistidine	Organic acids and derivatives	1.94	up	<0.05	2.69
Thioetheramide-PC	Undefined	1.87	up	<0.05	17.77
*N*-Acetyl-L-Histidine	Undefined	1.74	up	<0.05	3.00
1-hexadecanoyl-2-octadecadienoyl-sn-glycero-3-phosphocholine	Lipids and lipid-like molecules	1.74	up	<0.05	24.45
*N*-α-(tert-butoxycarbonyl)-l-histidine	Organic acids and derivatives	1.72	up	<0.05	3.84
3-dehydrocarnitine	Organic acids and derivatives	1.67	up	<0.05	1.14
PC(16:0/16:0)	Lipids and lipid-like molecules	1.60	up	<0.05	2.74
1,2-dilinoleoyl-sn-glycero-3-phosphoethanolamine	Lipids and lipid-like molecules	1.57	up	<0.05	1.81
*N*-α-acetyl-l-ornithine	Organic acids and derivatives	1.53	up	<0.05	1.32
L-Histidine	Organic acids and derivatives	1.45	up	<0.05	2.22
Pantothenic acid	Organic acids and derivatives	1.41	up	<0.05	1.45
1-Stearoyl-2-arachidonoyl-sn-glycerol	Lipids and lipid-like molecules	1.36	up	<0.05	4.39
1-Palmitoylglycerol	Undefined	1.27	up	<0.05	1.12
Inosine 5′-monophosphate	Nucleosides, nucleotides, and analogues	1.25	up	<0.05	5.66
1-hexadecyl-2-(8z,11z,14z-eicosatrienoyl)-sn-glycero-3-phosphocholine	Lipids and lipid-like molecules	0.79	down	<0.05	6.09
3-hydroxyanthranilic acid	Benzenoids	0.77	down	<0.05	5.70
2-oleoyl-1-stearoyl-sn-glycero-3-phosphoserine	Lipids and lipid-like molecules	0.76	down	<0.05	4.50
Adenosine	Nucleosides, nucleotides, and analogues	0.75	down	<0.05	3.31
1-hexadecyl-2-(5z,8z,11z,14z-eicosatetraenoyl)-sn-glycero-3-phosphocholine	Lipids and lipid-like molecules	0.73	down	<0.05	3.05
Betaine	Organic acids and derivatives	0.72	down	<0.05	15.85
1-(1z-octadecenyl)-2-(4z,7z,10z,13z,16z,19z-docosahexaenoyl)-sn-glycero-3-phosphocholine	Lipids and lipid-like molecules	0.66	down	<0.05	1.71
Pro-leu	Organic acids and derivatives	0.66	down	<0.05	13.01
Pheniramine	Organoheterocyclic compounds	0.61	down	<0.05	1.71
Carnitine	Organic nitrogen compounds	0.60	down	<0.05	12.08
Methyl (1-(cyclohexylmethyl)-1h-indole-3-carbonyl)-l-valinate	Organic acids and derivatives	0.57	down	<0.05	1.12
Palmitoyl sphingomyelin	Lipids and lipid-like molecules	0.53	down	<0.05	8.81
L-Alanine	Organic acids and derivatives	0.48	down	<0.05	1.99
Fenbuconazole	Undefined	0.41	down	<0.05	1.92

**Table 4:** Differential metabolites and major KEGG pathways.

Group	Classification	KEGG Pathway	Metabolite
Group H	Unsaturated fatty acids metabolism	Arachidonic acid metabolism	Pc(16:0/16:1)
		Linoleic acid metabolism	Pc(16:0/16:0)
		Alpha-linolenic acid metabolism	Pc(16:0/16:1)
	Amino acid metabolism	Phenylalanine, tyrosine and tryptophan biosynthesis	Anthranilic acid
		D-arginine and d-ornithine metabolism	Dl-arginine
		Arginine biosynthesis	*N*-alpha-acetyl-l-ornithine
		Histidine metabolism	L-histidine, Histamine, L-carnosine
		Beta-alanine metabolism	Histidine, L-carnosine, L-histidine, Pantothenic acid
	Reproduction pathway	GnRH signaling pathway	Diacylglycerol(18:0/20:4)
Group G	Energy metabolism	Glycolysis/gluconeogenesis	Pyruvate
		Citrate cycle (TCA cycle)	Pyruvate, Malate
		Pentose phosphate pathway	Pyruvate, Glyceric acid
		Pentose and glucuronate interconversions	Pyruvate
		Pyruvate metabolism	Pyruvate, Malate
		Carbon metabolism	Pyruvate, Malate, Glyceric acid, O-acetylserine, L-alanine
	Fatty acid synthesis and metabolism	Fatty acid biosynthesis	Myristic acid, Palmitic acid
		Fatty acid elongation	Palmitic acid
		Fatty acid degradation	Palmitic acid
		Biosynthesis of unsaturated fatty acids	Palmitic acid
		Fatty acid metabolism	Palmitic acid
		Glycerolipid metabolism	Glyceric acid

## Data Availability

The data relevant to the study are available from the corresponding author upon reasonable request. The data are not publicly available due to privacy.

## References

[1] FAOSTAT (2020). Food Supply Quantity of Food Balances.

[2] Baek K.H., Utama D.T., Lee S.G., An B.K., Lee S.K. (2016). Effects of Replacing Pork Back Fat with Canola and Flaxseed Oils on Physicochemical Properties of Emulsion Sausages from Spent Layer Meat. Asian-Australas. J. Anim. Sci..

[3] Karthik P., Kulkarni V.V., Sivakumar K. (2010). Preparation, storage stability and palatability of spent hen meal based pet food. J. Food Sci. Technol..

[4] Katemala S., Molee A., Thumanu K., Yongsawatdigul J. (2021). Meat quality and Raman spectroscopic characterization of Korat hybrid chicken obtained from various rearing periods. Poult. Sci..

[5] Chen Y., Qiao Y., Xiao Y., Chen H., Zhao L., Huang M., Zhou G. (2016). Differences in Physicochemical and Nutritional Properties of Breast and Thigh Meat from Crossbred Chickens, Commercial Broilers, and Spent Hens. Asian-Australas. J. Anim. Sci..

[6] Fan H., Bhullar K.S., Wu J. (2021). Spent Hen Muscle Protein-Derived RAS Regulating Peptides Show Antioxidant Activity in Vascular Cells. Antioxidants.

[7] Yu W., Field C.J., Wu J. (2018). Purification and identification of anti-inflammatory peptides from spent hen muscle proteins hydrolysate. Food Chem..

[8] Hong H., Fan H., Roy B.C., Wu J. (2021). Amylase enhances production of low molecular weight collagen peptides from the skin of spent hen, bovine, porcine, and tilapia. Food Chem..

[9] Kumar D., Tarafdar A., Dass S.L., Pareek S., Badgujar P.C. (2023). Antioxidant potential and amino acid profile of ultrafiltration derived peptide fractions of spent hen meat protein hydrolysate. J. Food Sci. Technol..

[10] Esparza Y., Bandara N., Ullah A., Wu J. (2018). Hydrogels from feather keratin show higher viscoelastic properties and cell proliferation than those from hair and wool keratins. Mater. Sci. Eng. C Mater. Biol. Appl..

[11] Zubair M., Wu J., Ullah A. (2019). Hybrid Bionanocomposites from Spent Hen Proteins. ACS Omega.

[12] Nicholson J.K., Lindon J.C., Holmes E. (1999). ‘Metabonomics’: Understanding the metabolic responses of living systems to pathophysiological stimuli via multivariate statistical analysis of biological NMR spectroscopic data. Xenobiotica.

[13] Zheng Y., Shi X., Hou J., Gao S., Chao Y., Ding J., Chen L., Qian Y., Shao G., Si Y. (2021). Integrating metabolomics and network pharmacology to explore Rhizoma Coptidis extracts against sepsis-associated acute kidney injury. J. Chromatogr. B Anal. Technol. Biomed. Life Sci..

[14] Talmor-Barkan Y., Bar N., Shaul A.A., Shahaf N., Godneva A., Bussi Y., Lotan-Pompan M., Weinberger A., Shechter A., Chezar-Azerrad C. (2022). Metabolomic and microbiome profiling reveals personalized risk factors for coronary artery disease. Nat. Med..

[15] Yoo S.A., Park S.E., Seo S.H., Lee H.J., Lee K.I., Son H.S. (2016). A GC-MS based metabolic profiling of fermented sausage supplemented with pineapple. Food Sci. Biotechnol..

[16] Lotfy M.M., Sayed A.M., AboulMagd A.M., Hassan H.M., El Amir D., Abouzid S.F., El-Gendy A.O., Rateb M.E., Abdelmohsen U.R., Alhadrami H. (2021). Metabolomic profiling, biological evaluation of Aspergillus awamori, the river Nile-derived fungus using epigenetic and OSMAC approaches. RSC Adv..

[17] Zhu G., Wang S., Huang Z., Zhang S., Liao Q., Zhang C., Lin T., Qin M., Peng M., Yang C. (2018). Rewiring of the Fruit Metabolome in Tomato Breeding. Cell.

[18] Tan C., Selamat J., Jambari N.N., Sukor R., Murugesu S., Khatib A. (2021). Muscle and Serum Metabolomics for Different Chicken Breeds under Commercial Conditions by GC-MS. Foods.

[19] Izquierdo-Garcia J.L., Rodriguez I., Kyriazis A., Villa P., Barreiro P., Desco M., Ruiz-Cabello J. (2009). A novel R-package graphic user interface for the analysis of metabonomic profiles. BMC Bioinform..

[20] Thevenot E.A., Roux A., Xu Y., Ezan E., Junot C. (2015). Analysis of the Human Adult Urinary Metabolome Variations with Age, Body Mass Index, and Gender by Implementing a Comprehensive Workflow for Univariate and OPLS Statistical Analyses. J. Proteome Res..

[21] Li J., Li Z., Ran J., Yang C., Lin Z., Liu Y. (2022). LC/MS-based lipidomics to characterize breed-specific and tissue-specific lipid composition of chicken meat and abdominal fat. LWT.

[22] Antonny B., Vanni S., Shindou H., Ferreira T. (2015). From zero to six double bonds: Phospholipid unsaturation and organelle function. Trends Cell Biol..

[23] Tasseva G., Bai H.D., Davidescu M., Haromy A., Michelakis E., Vance J.E. (2013). Phosphatidylethanolamine deficiency in Mammalian mitochondria impairs oxidative phosphorylation and alters mitochondrial morphology. J. Biol. Chem..

[24] Smokvarska M., Bayle V., Maneta-Peyret L., Fouillen L., Poitout A., Dongois A., Fiche J.B., Gronnier J., Garcia J., Höfte H. (2023). The receptor kinase FERONIA regulates phosphatidylserine localization at the cell surface to modulate ROP signaling. Sci. Adv..

[25] Hur S.J., Choi B.D., Choi Y.J., Kim B.G., Jin S.K. (2011). Quality characteristics of imitation crab sticks made from Alaska Pollack and spent laying hen meat. LWT—Food Sci. Technol..

[26] Choe J., Kim H.Y. (2020). Physicochemical characteristics of breast and thigh meats from old broiler breeder hen and old laying hen and their effects on quality properties of pressed ham. Poult. Sci..

[27] Jeong H.S., Utama D.T., Kim J., Barido F.H., Lee S.K. (2020). Quality comparison of retorted Samgyetang made from white semi-broilers, commercial broilers, Korean native chickens, and old laying hens. Asian-Australas. J. Anim. Sci..

[28] Bradbury K.E., Skeaff C.M., Green T.J., Gray A.R., Crowe F.L. (2010). The serum fatty acids myristic acid and linoleic acid are better predictors of serum cholesterol concentrations when measured as molecular percentages rather than as absolute concentrations. Am. J. Clin. Nutr..

[29] Harvey K.A., Walker C.L., Pavlina T.M., Xu Z., Zaloga G.P., Siddiqui R.A. (2010). Long-chain saturated fatty acids induce pro-inflammatory responses and impact endothelial cell growth. Clin. Nutr..

[30] Burdock G.A., Carabin I.G. (2007). Safety assessment of myristic acid as a food ingredient. Food Chem. Toxicol. Int. J. Publ. Br. Ind. Biol. Res. Assoc..

[31] Knottnerus S.J.G., Bleeker J.C., Wust R.C.I., Ferdinandusse S., IJlst L., Wijburg F.A., Wanders R.J.A., Visser G., Houtkooper R.H. (2018). Disorders of mitochondrial long-chain fatty acid oxidation and the carnitine shuttle. Rev. Endocr. Metab. Disord..

[32] Adeva-Andany M., Lopez-Ojen M., Funcasta-Calderon R., Ameneiros-Rodriguez E., Donapetry-Garcia C., Vila-Altesor M., Rodriguez-Seijas J. (2014). Comprehensive review on lactate metabolism in human health. Mitochondrion.

[33] Gray L.R., Tompkins S.C., Taylor E.B. (2014). Regulation of pyruvate metabolism and human disease. Cell. Mol. Life Sci..

[34] Qiu K., He W., Zhang H., Wang J., Qi G., Guo N., Zhang X., Wu S. (2022). Bio-Fermented Malic Acid Facilitates the Production of High-Quality Chicken via Enhancing Muscle Antioxidant Capacity of Broilers. Antioxidants.

[35] Yan E., Wang Y., He L., Guo J., Zhang X., Yin J. (2022). Effects of Dietary L-malic Acid Supplementation on Meat Quality, Antioxidant Capacity and Muscle Fiber Characteristics of Finishing Pigs. Foods.

[36] Liu Y., Guo X., Wang N., Lu S., Dong J., Qi Z., Zhou J., Wang Q. (2023). Evaluation of changes in egg yolk lipids during storage based on lipidomics through UPLC-MS/MS. Food Chem..

[37] Poccia D., Larijani B. (2009). Phosphatidylinositol metabolism and membrane fusion. Biochem. J..

[38] Rosenblat M., Vaya J., Shih D., Aviram M. (2005). Paraoxonase 1 (PON1) enhances HDL-mediated macrophage cholesterol efflux via the ABCA1 transporter in association with increased HDL binding to the cells: A possible role for lysophosphatidylcholine. Atherosclerosis.

[39] Shadan S., Holic R., Carvou N., Ee P., Li M., Murray-Rust J., Cockcroft S. (2008). Dynamics of lipid transfer by phosphatidylinositol transfer proteins in cells. Traffic.

[40] Niknafs S., Fortes M.R.S., Cho S., Black J.L., Roura E. (2022). Alanine-specific appetite in slow growing chickens is associated with impaired glucose transport and TCA cycle. BMC Genom..

[41] Peiretti P.G., Medana C., Visentin S., Giancotti V., Zunino V., Meineri G. (2011). Determination of carnosine, anserine, homocarnosine, pentosidine and thiobarbituric acid reactive substances contents in meat from different animal species. Food Chem..

[42] Boldyrev A.A., Stvolinsky S.L., Fedorova T.N., Suslina Z.A. (2010). Carnosine as a natural antioxidant and geroprotector: From molecular mechanisms to clinical trials. Rejuvenation Res..

[43] Sugiyama T., Tanaka H., Kawai S. (2000). Improvement of periarticular osteoporosis in postmenopausal women with rheumatoid arthritis by beta-alanyl-L-histidinato zinc: A pilot study. J. Bone Miner. Metab..

[44] Feehan J., Hariharan R., Buckenham T., Handley C., Bhatnagar A., Baba S.P., de Courten B. (2022). Carnosine as a potential therapeutic for the management of peripheral vascular disease. Nutr. Metab. Cardiovasc. Dis..

[45] McFarland G.A., Holliday R. (1994). Retardation of the senescence of cultured human diploid fibroblasts by carnosine. Exp. Cell Res..

[46] Suwanvichanee C., Sinpru P., Promkhun K., Kubota S., Riou C., Molee W., Yongsawatdigul J., Thumanu K., Molee A. (2022). Effects of β-alanine and L-histidine supplementation on carnosine contents in and quality and secondary structure of proteins in slow-growing Korat chicken meat. Poult. Sci..

[47] Dobkin-Bekman M., Naidich M., Pawson A.J., Millar R.P., Seger R., Naor Z. (2006). Activation of mitogen-activated protein kinase (MAPK) by GnRH is cell-context dependent. Mol. Cell. Endocrinol..

[48] Xu T., Gu L., Schachtschneider K.M., Liu X., Huang W., Xie M., Hou S. (2014). Identification of differentially expressed genes in breast muscle and skin fat of postnatal Pekin duck. PLoS ONE.

[49] Yu S., Wang G., Liao J., Shen X., Chen J., Chen X. (2023). Co-expression analysis of long non-coding RNAs and mRNAs involved in intramuscular fat deposition in Muchuan black-bone chicken. Br. Poult. Sci..

[50] Bee G., Biolley C., Guex G., Herzog W., Lonergan S.M., Huff-Lonergan E. (2006). Effects of available dietary carbohydrate and preslaughter treatment on glycolytic potential, protein degradation, and quality traits of pig muscles. J. Anim. Sci..

[51] Schilling M.W., Suman S.P., Zhang X., Nair M.N., Desai M.A., Cai K., Ciaramella M.A., Allen P.J. (2017). Proteomic approach to characterize biochemistry of meat quality defects. Meat Sci..

[52] Lee D., Lee H.J., Jung D.Y., Kim H.J., Jang A., Jo C. (2022). Effect of an animal-friendly raising environment on the quality, storage stability, and metabolomic profiles of chicken thigh meat. Food Res. Int..

[53] Mashek D.G., Coleman R.A. (2006). Cellular fatty acid uptake: The contribution of metabolism. Curr. Opin. Lipidol..

[54] Cui H., Liu L., Liu X., Wang Y., Luo N., Tan X., Zhu Y., Liu R., Zhao G., Wen J. (2022). A selected population study reveals the biochemical mechanism of intramuscular fat deposition in chicken meat. J. Anim. Sci. Biotechnol..

